# Efficacy of High-Dose Atorvastatin Loading in Patients With ST-Elevation Myocardial Infarction (STEMI) Undergoing Percutaneous Coronary Intervention: A Systematic Review of Randomized Controlled Trials

**DOI:** 10.7759/cureus.90817

**Published:** 2025-08-23

**Authors:** Mansi Yadav, Archana Dhami, Umar Ahsan, Laiba Nadeem, Fatima Raja, Muhammad Raif, Hussnain Mushtaq, Syed Momin Ali, Shafeen Bashir Butt, Nikhil Deep Kolanu

**Affiliations:** 1 Internal Medicine, Pandit Bhagwat Dayal Sharma Post Graduate Institute of Medical Sciences, Rohtak, IND; 2 Family Medicine, Avalon University School of Medicine, Willemstad, CUW; 3 Accident and Emergency, Epsom and St. Helier University Hospitals NHS Trust, London, GBR; 4 Medicine, King Edward Medical University, Lahore, PAK; 5 Internal Medicine, King Edward Medical University, Lahore, PAK; 6 Neurology, King Edward Medical University, Lahore, PAK; 7 Medicine and Surgery, King Edward Medical University, Lahore, PAK; 8 Internal Medicine, China Medical University, Shenyang, CHN

**Keywords:** atorvastatin, high-dose statin therapy, non-st-elevation myocardial infarction (nstemi), primary percutaneous coronary intervention, st-elevation myocardial infarction (stemi), systematic review

## Abstract

ST-segment elevation myocardial infarction (STEMI) remains a leading cause of cardiovascular mortality despite advances in primary percutaneous coronary intervention (PCI) techniques. High-dose statin loading has emerged as a potential cardioprotective strategy to optimize acute outcomes through pleiotropic effects beyond lipid reduction. This systematic review synthesizes evidence from randomized controlled trials examining the efficacy and safety of high-dose atorvastatin loading in STEMI patients undergoing primary PCI. A comprehensive literature search was conducted across major databases, including PubMed, Embase, Cochrane Library, and Scopus, from inception to May 2024. Five randomized controlled trials involving 559 patients met the inclusion criteria, comparing 80 mg atorvastatin loading with standard or no loading strategies. The evidence demonstrates significant benefits in reducing the no-reflow phenomenon and significant relative risk reduction. High-dose atorvastatin loading consistently reduced inflammatory markers, including high-sensitivity C-reactive protein and interleukin-6, while improving endothelial function parameters. Angiographic outcomes showed improvements in myocardial blush grade and corrected TIMI frame count. However, translation to hard clinical endpoints such as major adverse cardiovascular events showed inconsistent results across studies. The safety profile was reassuring, with no significant increases in hepatotoxicity, myopathy, or other statin-related adverse events. Despite promising mechanistic benefits, the heterogeneity in study designs, small sample sizes, and short follow-up periods limits definitive conclusions regarding optimal dosing strategies and patient selection criteria. Future large-scale trials with standardized protocols and long-term follow-up are needed to establish the clinical utility of high-dose atorvastatin loading in STEMI patients undergoing primary PCI.

## Introduction and background

ST-segment elevation myocardial infarction (STEMI) remains a leading cause of cardiovascular morbidity and mortality worldwide, with primary percutaneous coronary intervention (PCI) established as the gold standard treatment for timely reperfusion [1]. Despite significant advances in interventional techniques and adjunctive pharmacotherapy, optimal myocardial salvage and long-term outcomes remain challenging goals, particularly due to complications such as the no-reflow phenomenon, microvascular dysfunction, and reperfusion injury that can occur during primary PCI procedures [2].

Statins, specifically 3-hydroxy-3-methylglutaryl coenzyme A (HMG-CoA) reductase inhibitors, have demonstrated well-established benefits in cardiovascular disease prevention and management through their lipid-lowering properties and pleiotropic effects. Beyond cholesterol reduction, statins exhibit anti-inflammatory, antithrombotic, and endothelial-protective properties that may be particularly beneficial in the acute setting of STEMI [3]. The concept of statin loading, involving the administration of high-dose statin therapy immediately before or during PCI, has emerged as a potential strategy to maximize these acute cardioprotective effects [4]. High-dose atorvastatin may produce an optimal result for STEMI patients undergoing PCI by improving microvascular myocardial perfusion, as demonstrated in landmark trials such as the STATIN STEMI trial [5]. Loading-dose atorvastatin therapy before emergency PCI reduced the inflammatory response and myocardial dysfunction in these STEMI patients by lowering hs-CRP, BNP, and MMP-9. The mechanistic basis for these benefits involves multiple pathways, including stabilization of atherosclerotic plaques, reduction of inflammatory markers, improvement of endothelial function, and enhancement of nitric oxide bioavailability [6].

Recent meta-analysis has provided compelling evidence supporting the efficacy of high-dose statin loading strategies [7]. A high-dose loading of statins before PCI in patients with acute coronary syndrome (ACS) reduces major adverse cardiovascular and cerebrovascular events (MACCE) and reduces the risk of MI with no impact on mortality at 30 days [7]. Atorvastatin reduces MACCE in STEMI, while rosuvastatin reduces MACCE in non-ST-elevation myocardial infarction ACS (NSTE-ACS) at 30 days [7]. Nevertheless, immediate high-dose statin loading before PCI showed beneficial effects on myocardial perfusion, including significant reductions in the corrected thrombolysis in myocardial infarction (TIMI) flow grade count (p = 0.01), as well as increases in myocardial blush grade (p = 0.02) [8]. From a safety perspective, concerns regarding the acute administration of high-dose statins, particularly regarding hepatotoxicity, myopathy, and rhabdomyolysis, have been extensively evaluated. A clear dose-response relationship trial comparing the lowest (10 mg) and highest (80 mg) doses of atorvastatin found no significant differences in the incidence of myopathy/rhabdomyolysis, which was well below 0.1% with both doses. The acute loading strategy appears to have an acceptable safety profile, with most adverse events being mild and transient [9].

Despite these promising findings, several gaps remain in our understanding of optimal dosing strategies, timing of administration, patient selection criteria, and long-term outcomes. Although high-dose atorvastatin has been shown to reduce important patient outcomes such as MACE, there is still doubt that high-dose atorvastatin could have the same protective effects across all patient populations and clinical scenarios. Furthermore, the heterogeneity in study designs, dosing regimens, and outcome measures across published trials necessitates a comprehensive systematic evaluation. This systematic review aims to synthesize the current evidence regarding the safety and efficacy of atorvastatin loading before primary PCI in STEMI patients. By analyzing randomized controlled trials and observational studies, we seek to provide clinicians with evidence-based guidance on the optimal use of pre-procedural high-dose atorvastatin therapy while identifying areas requiring further investigation to improve patient outcomes in this high-risk population.

## Review

Materials and methods

This systematic review was conducted in accordance with the Preferred Reporting Items for Systematic Reviews and Meta-Analyses (PRISMA) guidelines, ensuring a structured and transparent approach to identifying, selecting, appraising, and synthesizing evidence regarding the impact of high-dose atorvastatin loading on clinical outcomes in patients with STEMI undergoing PCI [10].

Search Strategy

A comprehensive literature search was conducted across four major electronic databases: PubMed, Embase, Cochrane Library, and Scopus. The search strategy combined MeSH terms and relevant keywords, including "atorvastatin", "high-dose statin", "STEMI", "percutaneous coronary intervention", and "randomized controlled trials". Boolean operators ("AND", "OR") were applied to optimize the search. The search spanned from database inception to May 30, 2024, with language restricted to English. Reference lists of included articles and relevant reviews were also manually screened to identify additional eligible studies.

Eligibility Criteria

Studies were considered eligible for inclusion if they met the following criteria: they were randomized controlled trials (RCTs) involving adult patients aged 18 years or older who were diagnosed with STEMI and underwent PCI. Eligible studies specifically compared high-dose atorvastatin loading (typically 80 mg administered prior to PCI) with either a standard or no loading dose and reported on clinically relevant outcomes such as major adverse cardiovascular events (MACE), myocardial injury, infarct size, or all-cause mortality. Studies were excluded if they were observational in design, including case reports, reviews, editorials, or study protocols. In addition, trials that involved statins other than atorvastatin, lacked complete data or full-text availability, or were published in languages other than English were excluded from the review.

Data Extraction and Synthesis

Two independent reviewers screened titles and abstracts, followed by a full-text review to confirm eligibility. Discrepancies were resolved through discussion or consultation with a third reviewer. Key data points were extracted using a standardized form on Google Sheets, including author, publication year, study population, dosage regimen, timing of atorvastatin administration, primary and secondary outcomes, and duration of follow-up.

Data Analysis

Given the anticipated heterogeneity in outcome definitions and intervention timing across studies, a narrative synthesis was performed. Emphasis was placed on the consistency of findings, direction of effect, and magnitude of benefit in reducing periprocedural myocardial injury and post-PCI complications.

Quality Assessment

The methodological quality of included RCTs was independently assessed using the Newcastle-Ottawa Scale (NOS) adapted for randomized studies. The scale evaluates three core domains: selection of study groups, comparability of groups, and ascertainment of outcomes. Each study was rated on a scale of 0 to 9 stars. Studies scoring 7 or more stars were considered high quality. Discrepancies were resolved by consensus. This rigorous assessment ensures that conclusions drawn from the review are based on robust and reliable evidence.

Results

Study Selection Process

**Figure 1:**
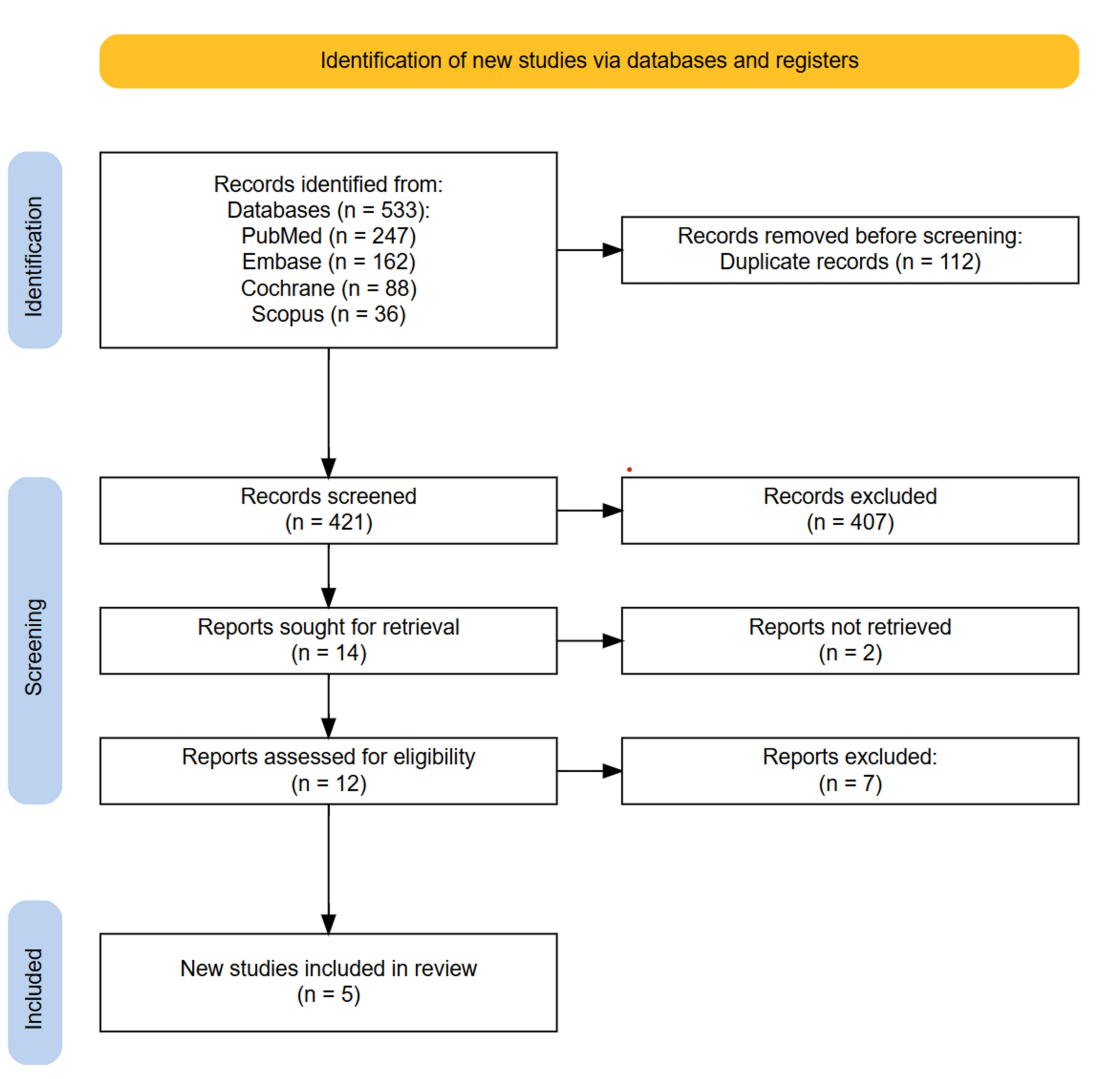
Preferred Reporting Items for Systematic Reviews and Meta-Analyses (PRISMA) diagram illustrating the study selection process.

A comprehensive systematic literature search was conducted across four major databases, namely, PubMed, Embase, the Cochrane Library, and Scopus, yielding a total of 533 articles. After the removal of 112 duplicates, 421 unique records remained. These were subjected to title and abstract screening, resulting in the exclusion of 407 articles. The full texts of the remaining 14 articles were assessed for eligibility, of which five RCTs met the inclusion criteria and were included in the final analysis. The process is depicted in the following PRISMA diagram (Figure 1).

Study Characteristics

The five included RCTs encompassed a total of 559 patients with STEMI undergoing primary percutaneous coronary intervention, representing diverse geographic populations from Mexico, Italy, China, and South Korea, with publication years ranging from 2010 to 2018. The patient populations were predominantly male (ranging from 75% to 90%), with mean ages between 54.5 and 61.5 years, reflecting typical STEMI demographics. All studies implemented similar inclusion criteria requiring adult patients (≥18 years) with a confirmed STEMI diagnosis and candidacy for primary PCI within 12 hours of symptom onset. The intervention protocols consistently employed 80 mg atorvastatin loading doses administered before primary PCI, although timing varied from emergency room administration to 90-150 minutes pre-procedure. Control groups received either standard-dose atorvastatin (10-20 mg daily) or no pre-procedural statin loading, with all studies implementing appropriate maintenance therapy post-PCI. Sample sizes ranged from 52 to 173 patients, with most studies employing 1:1 randomization ratios between intervention and control groups. The studies demonstrated methodological rigor with RCT designs, although follow-up periods varied considerably from 30 days to six months. Primary endpoints across studies included angiographic outcomes such as no-reflow phenomenon, myocardial blush grade, and corrected TIMI frame count, while secondary endpoints encompassed major adverse cardiovascular events, inflammatory markers, and endothelial function parameters. All studies reported comprehensive safety profiles with monitoring for hepatotoxicity, myopathy, and other statin-related adverse events, generally demonstrating acceptable tolerability profiles for high-dose atorvastatin loading strategies (Table 1).

**Table 1:** Characteristics and outcomes of randomized controlled trials evaluating high-dose atorvastatin loading in STEMI patients undergoing primary percutaneous coronary intervention. ARR: absolute risk reduction, AST: atorvastatin loading group, CI: confidence interval, CK-MB: creatine kinase-MB, cTFC: corrected TIMI frame count, eNOS: endothelial nitric oxide synthase, GP IIb/IIIa: glycoprotein IIb/IIIa, HR: hazard ratio, hs-CRP: high-sensitivity C-reactive protein, ICAM-1: intercellular adhesion molecule-1, IL-6: interleukin-6, LVEF: left ventricular ejection fraction, MACE: major adverse cardiovascular events, MBG: myocardial blush grade, NO: nitric oxide, NNT: number needed to treat, ox-LDL: oxidized low-density lipoprotein, PCI: percutaneous coronary intervention, RCT: randomized controlled trial, RH-PAT: reactive hyperemia peripheral arterial tonometry, SPECT: single-photon emission computed tomography, ST: standard treatment group, STEMI: ST-elevation myocardial infarction, TIMI: thrombolysis in myocardial infarction, TNF-α: tumor necrosis factor-alpha, UNL: upper normal limit

Author	Year	Country	Study design	Study period	Sample size	Population characteristics	Atorvastatin dose	Control group	Intervention details	Efficacy outcomes	Safety outcomes	Main findings	Rresults	Conclusion
García-Méndez et al. [11]	2018	Mexico	RCT	March 15, 2010 to August 10, 2011	103 patients (49 AST, 54 ST)	Adult patients (18-85 years) with acute STEMI within 12 hours of symptom onset, candidates for primary PCI	Loading dose: 80 mg before primary PCI. Maintenance: 40 mg daily for 30 days	Standard treatment alone is antiplatelet therapy (aspirin 300 mg + clopidogrel 300-600 mg), antithrombin therapy with heparin, ±GP IIb/IIIa inhibitors	80 mg atorvastatin loading dose administered 90-150 minutes before primary PCI in addition to standard treatment (AST group) vs. standard treatment alone (ST group)	No reflow: 27% vs. 63% (p < 0.0001) hs-CRP: 2.2 vs. 2.69 mg/dL (p = 0.72) IL-6: 5.2 vs. 6.35 pg/mL (p = 0.22)	MACE at 30 days: 47% vs. 57% (p = 0.28). Arrhythmias: 22% vs. 44% (p < 0.02)	No adverse events related to the experimental intervention reported	• 57% relative risk reduction for angiographic no reflow • 56% relative risk reduction for combined no reflow • Significant reduction in troponin I (17.1 vs. 30 ng/mL, p < 0.03) • Improved event-free survival for MACE (73.5% vs. 37%, p < 0.0001) • Independent predictor for no reflow prevention (HR 0.34, 95% CI 0.18-0.61, p < 0.001) • NNT: three patients • ARR: 36% angiographic, 39% combined no reflow	Administration of 80 mg atorvastatin loading dose before primary PCI is an effective strategy for preventing no reflow, improving clinical outcomes, and improving the event-free survival rate for major adverse cardiovascular events at 30 days in patients with acute STEMI
Gavazzoni et al. [12]	2017	Italy	RCT	February 2011 to February 2012	52 patients with STEMI	STEMI patients undergoing primary PCI; age: 58.4 ± 11 years; male: 88.4%; admitted within 48 hours of STEMI	High-dose: 80 mg daily (n = 26), moderate dose: 20 mg daily (n = 26)	Moderate-dose atorvastatin 20 mg daily	The first dose administered within 24 hours of first medical contact. Treatment duration: 30 days. Randomized 1:1 to receive either 80 mg or 20 mg atorvastatin daily	Primary endpoints: RH-PAT index (endothelial function), HS-CRP, IL6, TNFα, ox-LDL levels, and lipid profile changes	Not specifically detailed in the study	After one month: High-dose vs. moderate-dose showed significant differences in:• HS-CRP: 0.04 ± 0.02 vs. 0.36 ± 0.3 mg/dL (P = 0.001) • IL6: 1.12 ± 0.93 vs. 3.13 ± 2.84 pg/mL (P = 0.03) • RH-PAT index: 1.96 ± 0.16 vs. 1.72 ± 0.19 (P = 0.002) • Greater reduction in TNFα and ox-LDL in the high-dose group	High-dose atorvastatin showed superior vascular protective effects compared to moderate dose, with better improvement in endothelial function and greater reduction in inflammatory markers.	Higher-dose statin therapy in STEMI patients undergoing primary PCI showed earlier and greater vascular protective effects than moderate dose. High-dose treatment resulted in better endothelial function improvement and more significant anti-inflammatory effects in the vulnerable post-discharge period.
Yong et al. [13]	2014	China	RCT	October 2010 to June 2011	60 patients	STEMI patients undergoing primary PCI. Age: 54.5 ± 12.7 (loading), 61.5 ± 11.7 (regular), 55.6 ± 7.8 (control). Male: 90%, 75%, and 80%, respectively. Diabetes: 20%, 10%, 25%. Hypertension: 60%, 60%, 55%. Current smokers: 75%, 70%, 85%	Loading dose group: 80 mg prior to PCI. Regular dose group: 20 mg prior to PCI. Post-PCI: All groups received 20 mg/day.	Control group without atorvastatin prior to PCI (n = 20)	Primary PCI within 12 hours of symptom onset. Aspirin 300 mg + Clopidogrel 300-600 mg pretreatment. Weight-adjusted heparin. GP IIb/IIIa inhibitors as per the surgeon's discretion. Standard post-PCI therapy	Primary: endothelial function (eNOS, NO), inflammatory markers (IL-6, TNF-α, ICAM-1). Secondary: peak CK-MB, ST-segment resolution, LVEF, six-month MACEs	Liver function monitoring, myalgia assessment, no significant liver function differences, no myalgia reported in any group	eNOS: The regular-dose group had significantly higher levels immediately and 24-hour post-PCI IL-6. The loading dose group had significantly lower pre-PCI levels of NO, TNF-α, and ICAM-1. No significant differences in clinical outcomes. No significant differences in CK-MB, ST-resolution, LVEF, or MACEs.	• eNOS immediately post-PCI: 12.73 ± 3.22 vs. 10.26 ± 3.35 vs. 10.19 ± 3.93 (p = 0.026) • eNOS 24-hour post-PCI: 13.86 ± 1.33 vs. 12.28 ± 1.24 vs. 12.74 ± 1.46 (p = 0.002) • IL-6 pre-PCI: 90.77 ± 7.65 vs. 95.59 ± 4.27 vs. 94.32 ± 3.69 (p = 0.023) • MACEs: 10% vs. 10% vs. 15% (p > 0.05) • Peak CK-MB: 240 vs. 209 vs. 246 U/l (p = 0.558)	Atorvastatin loading in patients with STEMI undergoing primary PCI may not have protective effects on endothelial function and inflammatory reaction. The damage in STEMI may be too severe for a single dose of atorvastatin to elicit improvement in the short timeframe (~1.2 hours).
Hahn et al. [14]	2011	South Korea	RCT	August 2007 to February 2009	173 patients (89 atorvastatin, 84 control) with valid SPECT data	STEMI patients undergoing primary PCI within 12 hours. Mean age: 55.5 ± 12.1 vs. 59.7 ± 12.8 years. Male: 85.4% vs. 82.1%	80 mg before PCI, continued for five days, then reduced to 10 mg/day	10 mg atorvastatin daily starting the day after primary PCI	First dose administered: As early as possible before primary PCI. Treatment duration: five days of high-dose treatment, then reduced to 10 mg daily. Randomized: 1:1 to receive either 80 mg atorvastatin before PCI and continued for five days, then 10 mg daily, or 10 mg atorvastatin daily starting the day after PCI.	Myocardial blush grade, complete ST resolution at 60 minutes; major adverse cardiac events at six months	ALT elevation >3x UNL: 10.1% vs 7.1% (P = 0.49). Dose reduction needed in 6.7% of atorvastatin patients	No significant difference in infarct size: 22.2% ± 15.5% vs. 21.6%± 15.4% (P = 0.79). Median: 19.0% vs. 18.0% (P = 0.76)	No differences in myocardial blush grade 2/3 (72.8% vs. 81.9%, P = 0.33) or complete ST resolution (43.2% vs. 47.5%, P = 0.57). MACE at six months: 7.9% vs. 13.1% (P = 0.26)	Pretreatment with high-dose atorvastatin (80 mg) followed by five-day maintenance therapy did not reduce infarct size measured by SPECT in STEMI patients undergoing primary PCI. Well-tolerated but ineffective for cardioprotection
Kim et al. [5]	2010	South Korea	RCT	March 2007 to December 2008	171 patients (86 high-dose, 85 low-dose)	STEMI patients; mean age 60.3 ± 11.4 years; 77.2% male; pain-to-balloon ~231-241 min	High-dose: 80 mg vs. low-dose: 10 mg atorvastatin pre-treatment	10 mg atorvastatin pre-treatment (n = 85)	The first dose was administered in the emergency room before primary PCI. Treatment duration was a single loading dose before PCI, then 10 mg daily maintenance, with both groups receiving identical post-PCI treatment	cTFC, MBG, ST-segment resolution at 90 min post-PCI	Adverse events, stent thrombosis, mortality	No significant MACE difference (5.8% vs. 10.6%, p = 0.26); improved flow parameters in the high-dose group	Improved coronary flow and microvascular perfusion but no MACE reduction	Larger studies needed to confirm clinical benefits

Quality Assessment

The methodological quality of included RCTs was systematically evaluated using the Newcastle-Ottawa Scale adapted for randomized studies, assessing selection of study groups, comparability of groups, and outcome ascertainment. Two independent reviewers conducted quality assessments, with discrepancies resolved through consensus discussion. The overall quality was high across all five included studies, with scores ranging from seven to eight stars out of a maximum of nine stars (Table 2). All studies demonstrated adequate randomization procedures and appropriate patient selection criteria. Comparability between intervention and control groups was well-maintained across studies, with baseline characteristics appropriately balanced. Outcome ascertainment was generally robust, with most studies employing objective angiographic and laboratory measurements. The primary limitations identified included potential selection bias in some studies due to a single-center design and a lack of blinding in outcome assessment for certain endpoints. However, the use of objective laboratory and angiographic outcomes minimized the impact of assessment bias. The high-quality ratings support the reliability of findings and strengthen the evidence base for clinical decision-making regarding high-dose atorvastatin loading in STEMI patients.

**Table 2:** Quality assessment of included studies using the Newcastle-Ottawa Scale. ★ = 1 point; maximum possible score = 9 points; high quality = ≥7 points

Author	Selection	Comparability	Outcomes	Total score	Quality rating
García-Méndez et al. [11]	★★★★	★★	★★	8/9	High
Gavazzoni et al. [12]	★★★★	★★	★★	8/9	High
Yong et al. [13]	★★★	★★	★★	7/9	High
Hahn et al. [14]	★★★★	★★	★★	8/9	High
Kim et al. [5]	★★★	★★	★★	7/9	High

Discussion

The systematic review of RCTs examining high-dose atorvastatin loading in STEMI patients undergoing primary PCI reveals compelling evidence supporting the cardioprotective benefits of this therapeutic strategy, although outcomes vary across different clinical endpoints and patient populations. The most consistent and clinically significant finding was the substantial reduction in no-reflow phenomenon, with Méndez et al. demonstrating a remarkable 57% relative risk reduction (27% vs. 63%, p < 0.0001) in patients receiving 80 mg atorvastatin loading compared to standard treatment [11]. This finding is particularly important given that the no-reflow phenomenon represents a major determinant of clinical outcomes in STEMI patients, affecting both short-term myocardial salvage and long-term cardiovascular prognosis. The mechanistic basis for this benefit likely involves multiple pathways, including acute anti-inflammatory effects, endothelial protection, and improvement in microvascular function, as evidenced by the significant reductions in inflammatory markers such as high-sensitivity C-reactive protein and interleukin-6 observed across multiple studies [15].

The anti-inflammatory effects of high-dose atorvastatin loading appear to be both rapid and clinically meaningful, with Gavazzoni et al. demonstrating superior inflammatory marker reduction in the high-dose group compared to moderate-dose therapy (HS-CRP: 0.04 ± 0.02 vs. 0.36 ± 0.3 mg/dL, p = 0.001) [12]. These findings align with the pleiotropic effects of statins beyond lipid-lowering, suggesting that the acute cardioprotective benefits may be mediated through rapid stabilization of atherosclerotic plaques, reduction in inflammatory cascade activation, and improvement in endothelial function [7-12]. The endothelial protective effects were further supported by improved reactive hyperemia peripheral arterial tonometry (RH-PAT) index scores, indicating enhanced endothelial function in patients receiving high-dose atorvastatin loading.

However, the translation of these mechanistic benefits into hard clinical endpoints presents a more complex picture. While some studies demonstrated trends toward reduced major adverse cardiovascular events, the statistical significance and magnitude of these effects were inconsistent across trials. Hahn et al. found no significant difference in myocardial infarct size measured by SPECT imaging (22.2% ± 15.5% vs. 21.6% ± 15.4%, p = 0.79), suggesting that the acute loading strategy may not universally translate into myocardial salvage benefits [14]. Similarly, Kim et al. reported improved coronary flow parameters but no significant reduction in major adverse cardiovascular events (5.8% vs. 10.6%, p = 0.26), highlighting the complexity of translating surrogate endpoints into clinical outcomes [5].

The safety profile of high-dose atorvastatin loading appears reassuring across all included studies, with no significant increases in hepatotoxicity, myopathy, or other statin-related adverse events. This finding is particularly important given concerns about acute high-dose statin administration in the setting of acute myocardial infarction, where patients may have compromised hepatic or renal function. The low incidence of adverse events supports the feasibility of implementing high-dose atorvastatin loading strategies in clinical practice, provided appropriate monitoring protocols are established.

The heterogeneity in study designs, patient populations, and outcome measures across trials limits the ability to draw definitive conclusions about optimal dosing strategies and patient selection criteria. The timing of atorvastatin administration varied considerably, from emergency room administration to pre-procedural loading, potentially affecting the magnitude of observed benefits. Additionally, the relatively small sample sizes and short follow-up periods in most studies limit the power to detect differences in hard clinical endpoints such as mortality or long-term cardiovascular events.

Limitations and Future Directions

This systematic review has several important limitations that should be considered when interpreting the findings and their clinical implications. The relatively small sample sizes across included studies, ranging from 52 to 173 patients, limit the statistical power to detect clinically meaningful differences in major adverse cardiovascular events and mortality outcomes. The heterogeneity in study designs, particularly regarding the timing of atorvastatin administration (ranging from emergency room to 90-150 minutes pre-procedure), dosing protocols, and outcome definitions, precludes definitive meta-analysis and limits the generalizability of findings. Geographic and ethnic diversity across studies, while representing broader populations, introduces potential confounding variables related to genetic polymorphisms affecting statin metabolism and cardiovascular risk profiles. The short follow-up periods in most studies (30 days to six months) prevent assessment of long-term cardiovascular outcomes and the durability of observed benefits.

Future research should focus on large-scale, multinational RCTs with standardized protocols for atorvastatin loading timing, dosing, and outcome measurement. Long-term follow-up studies are essential to determine whether the acute benefits of high-dose atorvastatin loading translate into sustained improvements in cardiovascular outcomes and mortality. Investigation of optimal patient selection criteria, including consideration of baseline cardiovascular risk factors, concomitant medications, and genetic factors affecting statin response, would enhance the precision of this therapeutic approach. Additionally, economic analyses comparing the cost-effectiveness of high-dose atorvastatin loading strategies with standard care would provide valuable information for healthcare policy decisions and clinical implementation guidelines.

## Conclusions

This systematic review of five RCTs involving 559 STEMI patients demonstrates that high-dose atorvastatin loading (80 mg) before primary PCI provides significant cardioprotective benefits, particularly in reducing the no-reflow phenomenon and improving inflammatory markers. The intervention consistently reduced high-sensitivity C-reactive protein and interleukin-6 levels while enhancing endothelial function parameters. Angiographic outcomes showed improvements in myocardial blush grade and corrected TIMI frame count. However, translation to hard clinical endpoints such as major adverse cardiovascular events yielded inconsistent results across studies. The safety profile was reassuring with no significant increases in hepatotoxicity or myopathy. Despite promising mechanistic benefits, the heterogeneity in study designs, small sample sizes, and short follow-up periods limits definitive conclusions. Future large-scale trials with standardized protocols and long-term follow-up are essential to establish optimal dosing strategies and patient selection criteria for high-dose atorvastatin loading in STEMI patients undergoing primary PCI.
